# Supplementary material for: Automatic detection and decoding of honey bee waggle dances
Source: PLoS One. 2017 Dec 13;12(12):e0188626. doi: 10.1371/journal.pone.0188626 (PMC5728493; doi:10.1371/journal.pone.0188626)
Supplement: S1 Text — This document contains further information on the recording setup, with an emphasis on technical details. (PDF) [file pone.0188626.s001.pdf]

Specifications of the recording setup used during reported experiments

Cameras

We use Sony PlayStation PS3Eye camera[1]. To run the camera on a PC and modify the native frame rate of the camera it is necessary to use the third-party driver CL Eye Platform Driver developed by Code Laboratories[2].

Any camera capable to stream at QVGA @ 100 fps or higher should suffice to generate video sequences where waggle runs are discernable.

Lighting

If dances on both sides of the comb are to be detected, both sides need to be illuminated, this, however, reduces contrast in both cameras because some light will pass through the comb and enter the other side's camera. So, observing just one side will improve image quality and in effect reduce false positive detections.

We recorded the activity of the colony day and night, to avoid perturbing the colony, we use IR LED clusters[3] with a wavelength of 840 nm. In general, any constant source of light under which bees are discernable from the background suffices for the correct performance of the software modules. The entire structure was enveloped with a highly IR reflective foil with small embossments for light dispersion. The IR LED clusters pointed towards the foil to create a homogeneous ambient lighting and reduce reflections on the glass panes. To make the PS3Eye camera sensitive to IR light, we removed the built-in IR filter.

---

[1] More info here: https://en.wikipedia.org/wiki/PlayStation_Eye
[2] Available here: https://codelaboratories.com/products/eye/driver/
[3] We used an array of 22 clusters per side (TV6700 from Abus).
