# Supplementary material for: Automatic detection and decoding of honey bee waggle dances
Source: PLoS One. 2017 Dec 13;12(12):e0188626. doi: 10.1371/journal.pone.0188626 (PMC5728493; doi:10.1371/journal.pone.0188626)
Supplement: S2 Text — This document contains diagrams and detailed information on the functioning of the software modules. (PDF) [file pone.0188626.s002.pdf]

Further details on the software modules.

Our software consists of four modules that are executed in sequence. All modules can either run offline on video recordings or online on live video streaming.

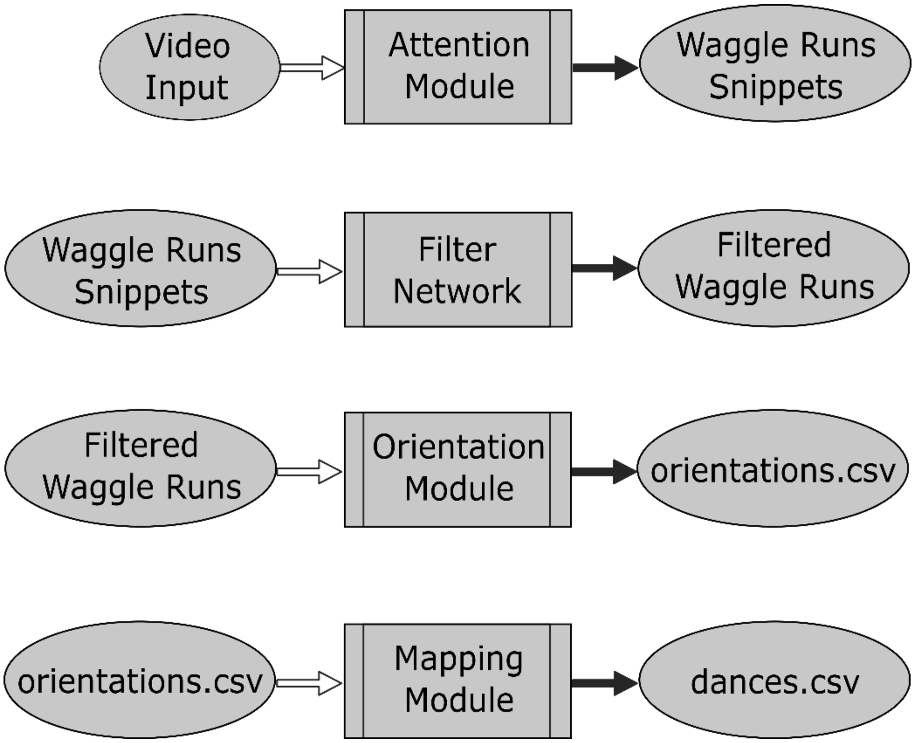

Fig 1. Software modules. The four modules that constitute our system can run offline in sequence.

Attention Module (AM)

The attention module (AM) is the first element of our system; it usually runs in real-time, this way, only snippets of the potential waggle runners are saved to disk instead of all the honeycomb surface, saving a significant amount of storing space.

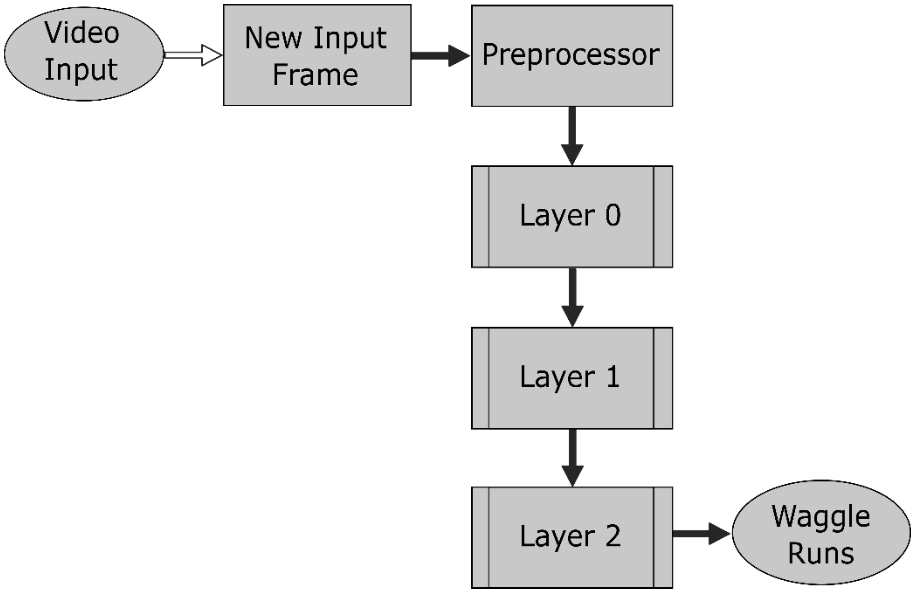

Fig 2. Flowchart of the attention module. Each new input frame is preprocessed before its pixel's values are used to update the DDs' score. If after undergoing all three layers of the module a waggle run is detected, 50 x 50 px images snippets of the WR sequence are stored to disk.

Filter Network (FN)

A 3D convolutional network was trained to process the sequence of 50 x 50 px. The scalar output of the net is then thresholded to predict if the sequence contains a waggling bee.

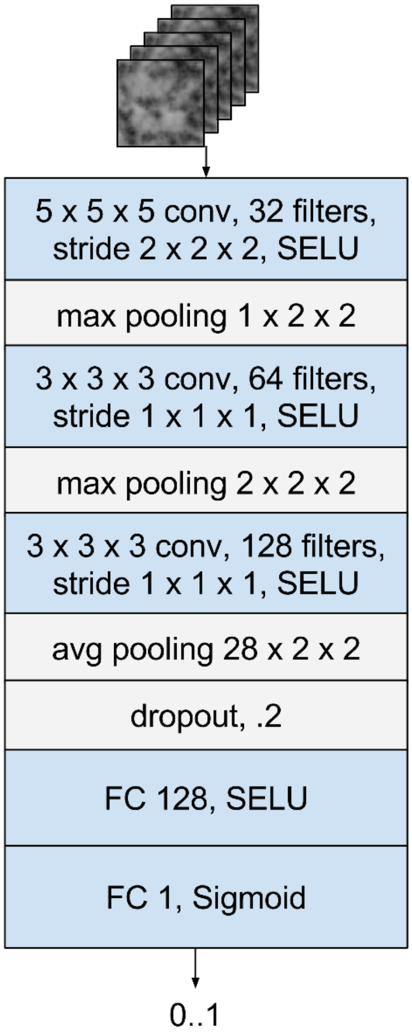

Fig 3. ConvNet structure. The raw sequences of images are processed by two stacked 3D convolution layers with SELU nonlinearities. The outputs of the second convolutional layer are flattened using average pooling in all three dimensions. A final fully connected layer with a sigmoid nonlinearity computes the probability of the sequence being a dance or not. Dropout is applied after the average pooling operation to reduce overfitting.

Orientation Module (OM)

Dancing bees waggle at a frequency of about 13 Hz. Images resulting from subtracting consecutive frames depicting a waggling bee exhibit a Gabor filter-like pattern which orientation is aligned to the bee's body. While the location of this pattern depends on the location of the dancing bee, its Fourier transformation provides a location-independent representation and preserves information of the bee's orientation.

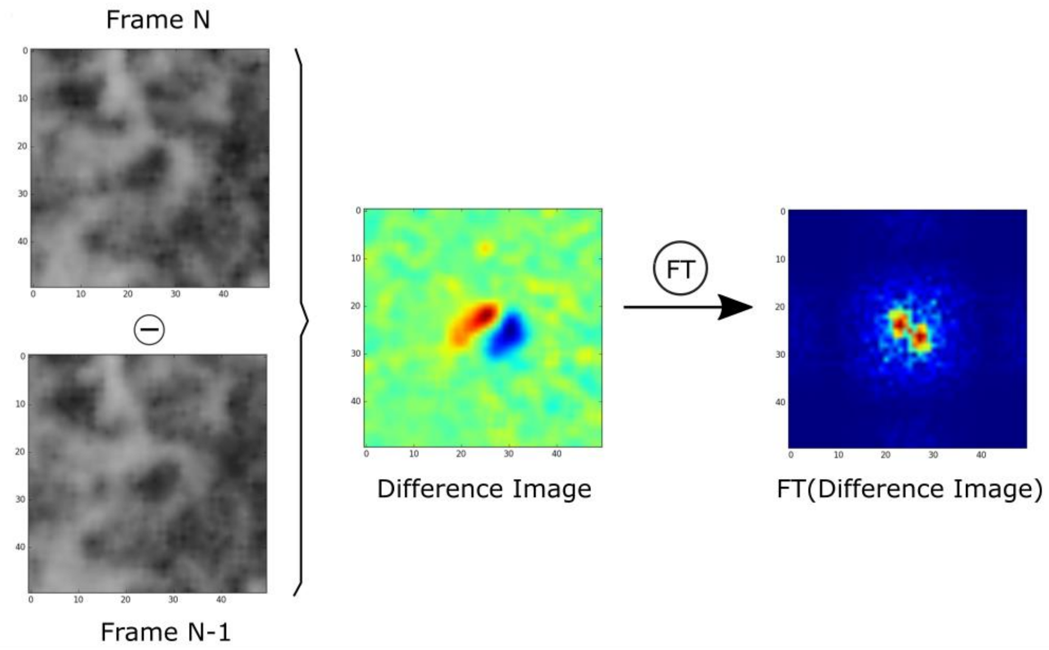

Fig 4. Difference image and its Fourier transformation. The difference image exhibits a Gabor filter-like pattern. The Fourier transformation of this image provides a location independent representation of the waggle event while preserving information of the bee's orientation.

Mapping Module (MM)

The mapping module (MM) reads the output of the AM and OM. The waggle runs are clustered using a hierarchical agglomerative clustering (HAC) approach.

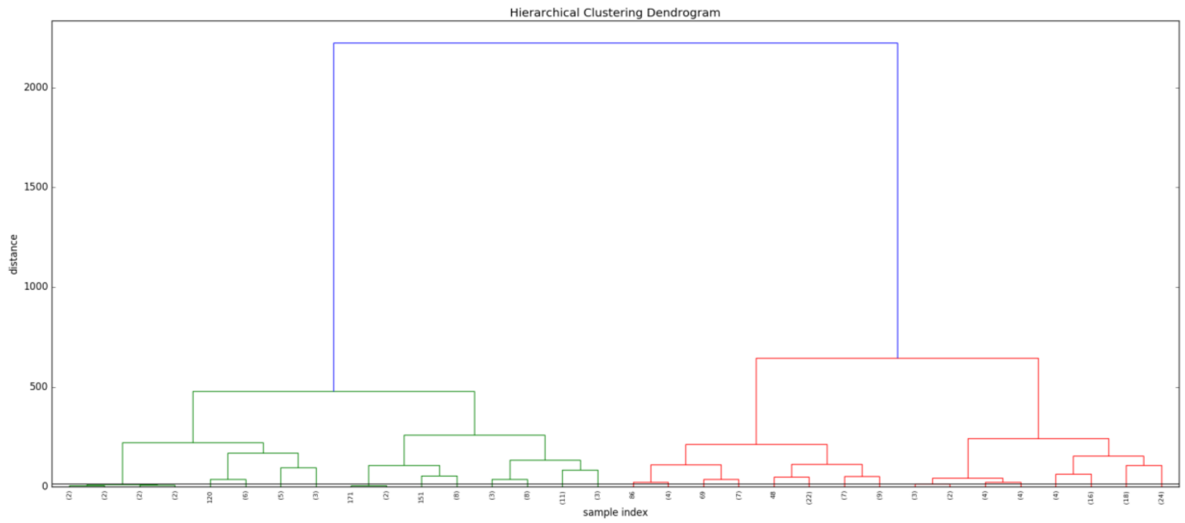

Fig 5. A simplified dendrogram depicting the clustering of 200 waggle runs. In this simplified version, only the last four levels of the dendrogram are displayed.

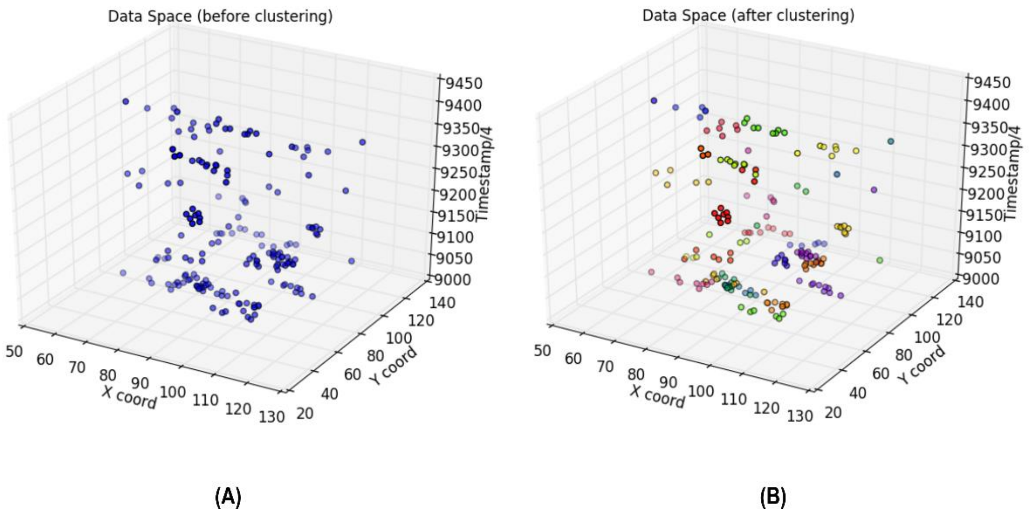

Fig 6. Depiction of 200 waggle runs in the data space XYT. (A) A set of 200 WRs plotted in the data space XYT, where values in the axes X and Y correspond to their comb location, and values in axis T to their time of occurrence. (B) Using an HAC approach, those WRs within a maximum Euclidean distance of d_{max3} are clustered together and considered as belonging to the same dance.

Links
All data and code used in these experiments is publicly available at (https://github.com/BioroboticsLab/WDD_paper)

To estimate the Solar azimuth and elevation at the moment of the dance, we use a Matlab script written by Darin Koblick and available in File Exchange (https://de.mathworks.com/matlabcentral/fileexchange/23051-vectorized-solar-azimuth-and-elevation-estimation?focused=3784972&tab=function)
