# Supplementary material for: Automatic detection and decoding of honey bee waggle dances
Source: PLoS One. 2017 Dec 13;12(12):e0188626. doi: 10.1371/journal.pone.0188626 (PMC5728493; doi:10.1371/journal.pone.0188626)
Supplement: S3 Text — This document provides additional results supporting the case of study presented in the experimental validation and results section. (PDF) [file pone.0188626.s003.pdf]

Error distributions at multiple levels of analysis.

To evaluate the performance of the Orientation Module (OM), we first manually defined angles for a 200 waggle runs data set. The angles were measured by eight human observers using a custom user interface (Fig 1), users had to trace the line that best fitted the dancer's body.

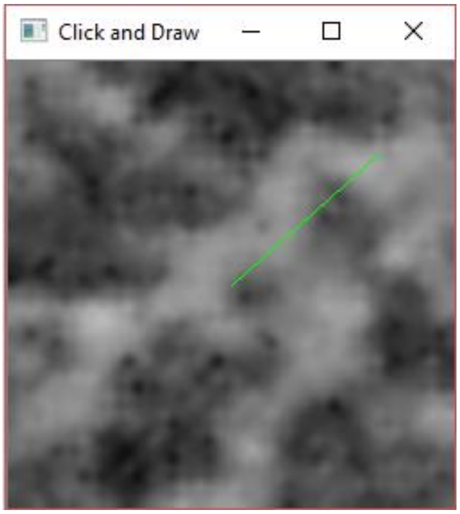

Fig 1. Screen capture of the GT generator.

The reference angle for each Waggle Run (WR) is defined as the average of the eight manually extracted angles. The human generated data has an SD of 6.66° (Fig 2).

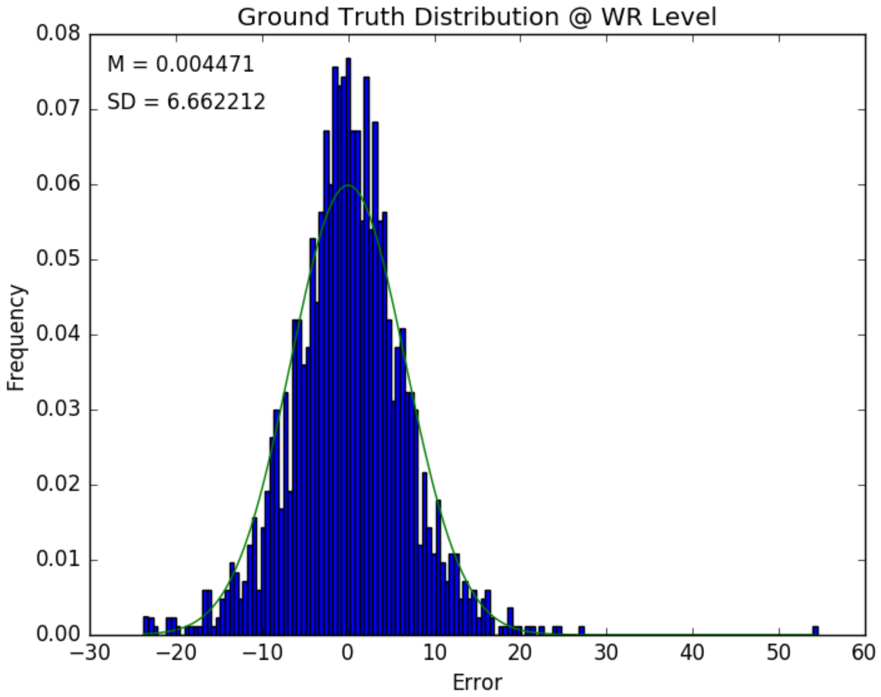

Fig 2. Ground truth distribution @ WR level.

Applying the Mapping Module (MM) to the 200 WRs data set we identified a total of 53 dances, only 23 of them composed of 4 or more waggle runs. An average angle was computed for the waggle dance and each user. The error distribution of the ground truth at the dance level has an SD of 3.67° (Fig 3).

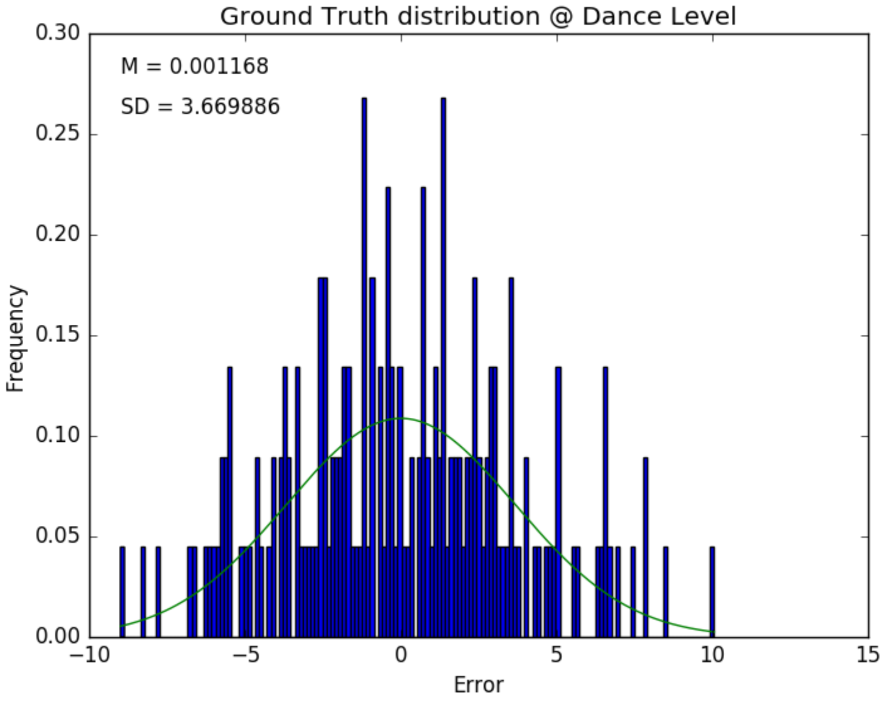

Fig 3. Ground truth distribution @ Dance level.

The OM obtains the orientation of the dancer through Principal Component Analysis (PCA). As a result of this process we end up with two possible waggle directions, unfortunately, the method implemented in the OM to disambiguate between these two directions only work for about 90% of the WRs. Considering the angles computed for the 200 WRs with the OM, we obtained an average error of -5.18° (SD = 53.18°) (Fig 4).

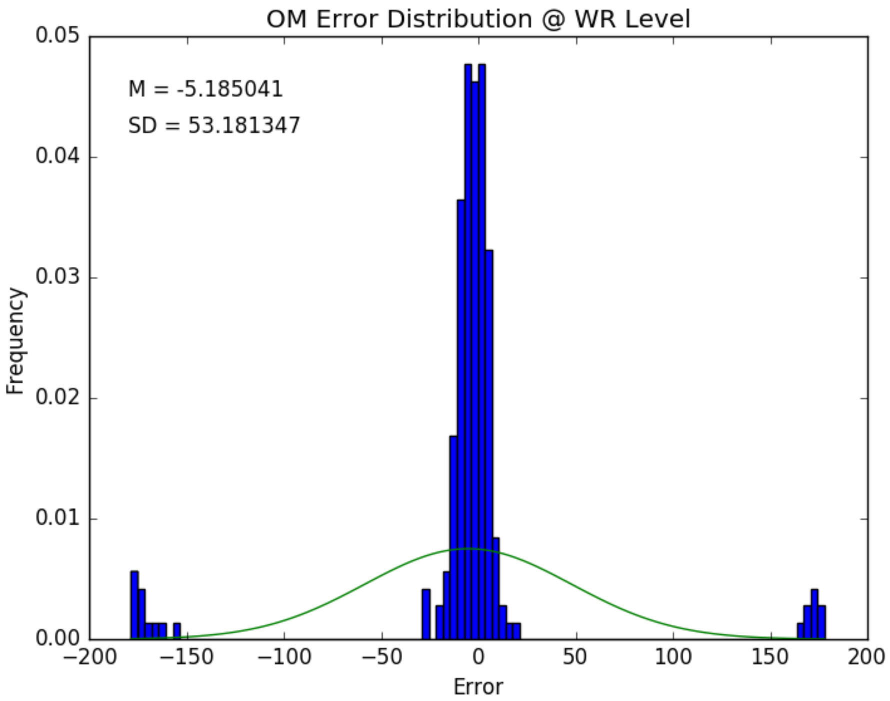

Fig 4. OM's error distribution @ WR level.

Manually removing the WRs that were incorrectly disambiguated, we obtain a mean value of -2.92° with an SD of 7.37° (Fig 5).

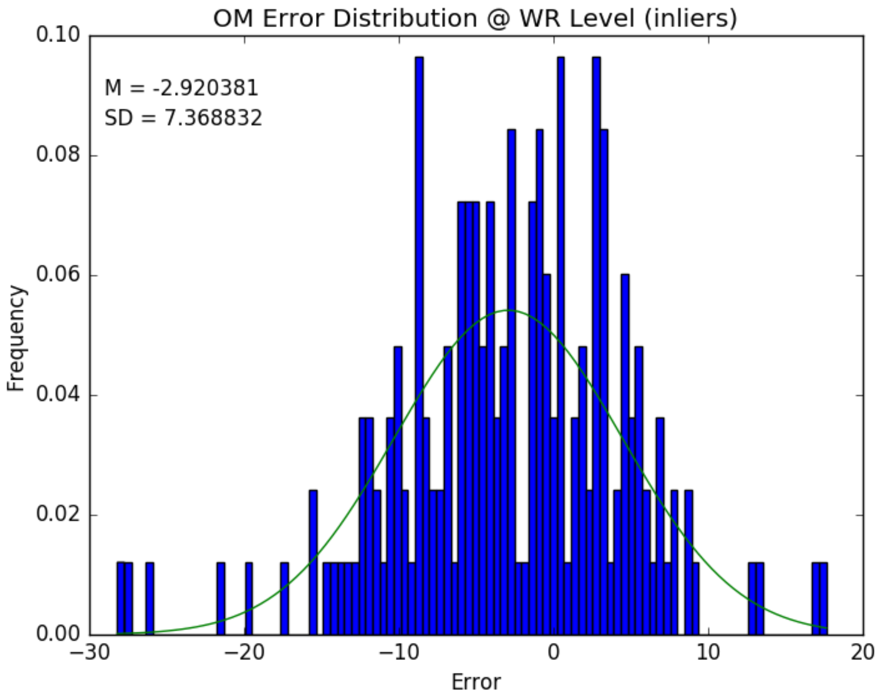

Fig 5. OM's error distribution @ WR level (inliers).

We also evaluated the performance of the system at the dance level. Using the Mapping Module (MM), we clustered the WRs in dances and removed the outliers. As a result we obtained an error distribution with a mean of -3.27° and an SD of 5.48°.

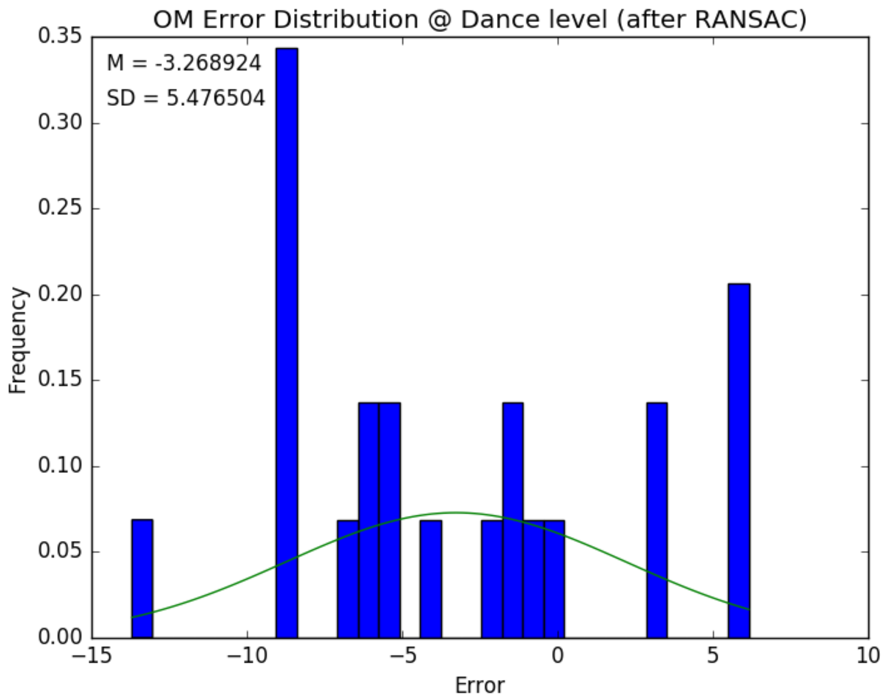

Fig 6. OM’s error distribution @ Dance level (after RANSAC)

Once the outliers have been removed we can compute a new error distribution at the WR level, this time with a mean value of -2.02° and an SD of 6.13°, well within the SD observed in the human defined angles.

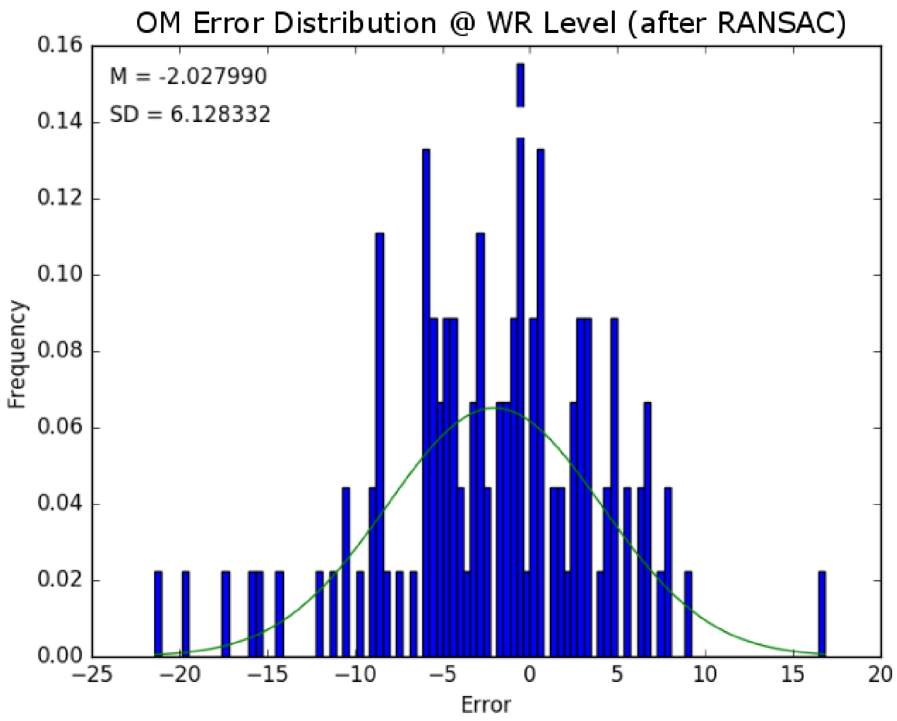

Fig 7. OM's error distribution @ Dance level (after RANSAC)

Links

All data and code used in these experiments are publicly available at (https://github.com/BioroboticsLab/WDD_paper)
