# Supplementary figures and images for: Automatic detection and decoding of honey bee waggle dances
Source: PLoS One. 2017 Dec 13;12(12):e0188626. doi: 10.1371/journal.pone.0188626 (PMC5728493; doi:10.1371/journal.pone.0188626)

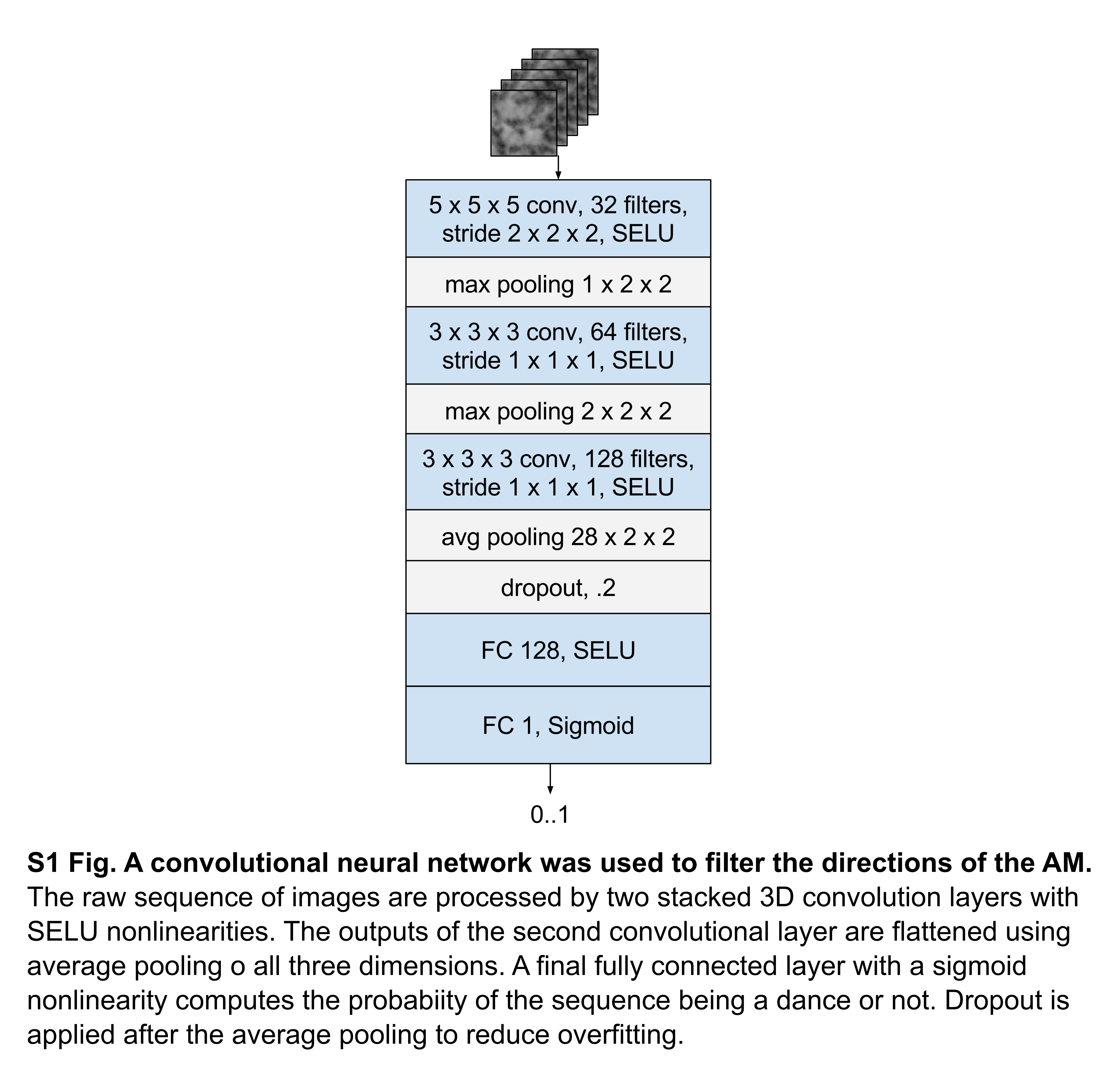

Supplement: S1 Fig — The raw sequences of images are processed by two stacked 3D convolution layers with SELU nonlinearities. The outputs of the second convolutional layer are flattened using average pooling on all three dimensions. A final fully connected layer with a sigmoid nonlinearity computes the probability of the sequence being a dance or not. Dropout is applied after the average pooling operation to reduce overfitting. (PNG) [file pone.0188626.s004.png]

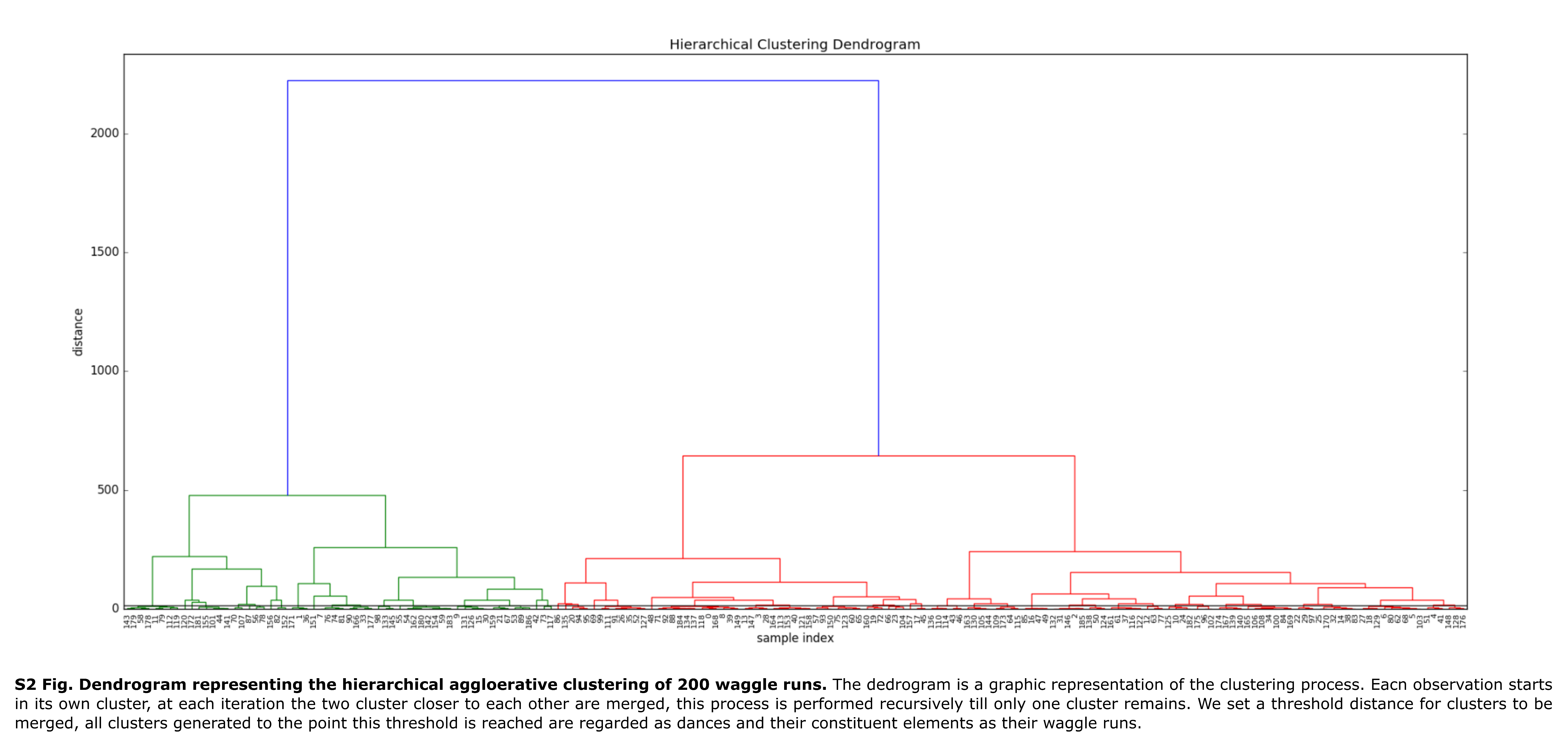

Supplement: S2 Fig — The dendrogram is a graphic representation of the clustering process. Each observation starts it its own cluster, at each iteration the two clusters closer to each other are merged, this process is performed recursively till only one cluster remains. We set a threshold distance for clusters to be merged, all clusters generated to the point this threshold is reached are regarded as dances and their constituent elements as their waggle runs. (PNG) [file pone.0188626.s005.png]

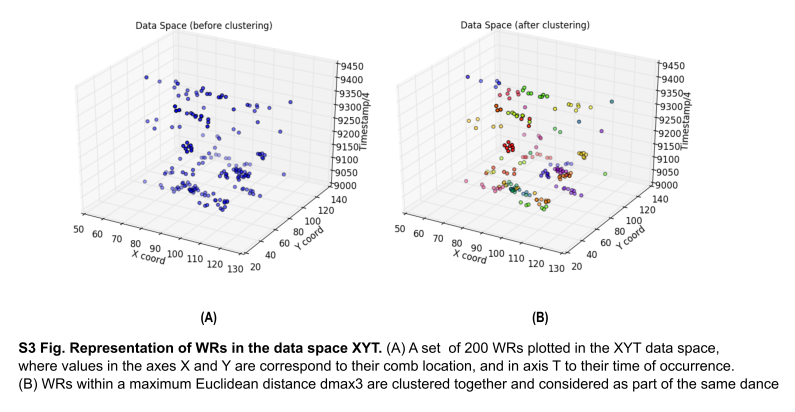

Supplement: S3 Fig — (A) Representation of a set of 200 WRs in the XYT data space, where values in the axes X and Y are defined by their comb location, and in axis T by their time of occurrence. (B) WRs within a maximum Euclidean distance of dmax3 are clustered together and regarded as dances. (PNG) [file pone.0188626.s006.png]
